# Pre-Hospital Critical Care for Out-of-Hospital Cardiac Arrests with Return of Spontaneous Circulation: A Retrospective Observational Study

**DOI:** 10.3390/jcm14030966

**Published:** 2025-02-03

**Authors:** Adam J. R. Watson, Delphi Henderson, Ryan Beecham, James Ward, Peter Owen, Julian Hannah, James Plumb, Ahilanandan Dushianthan

**Affiliations:** 1 University Hospital Southampton NHS Foundation Trust, Southampton SO16 6YD, UK; 2 Clinical & Experimental Sciences, Faculty of Medicine, University of Southampton, Southampton SO16 6YD, UK; 3 Hampshire & Isle of Wight Air Ambulance, Southampton SO16 0BT, UK; 4 Perioperative & Critical Care Theme, NIHR Southampton Biochemical Research Centre, Southampton SO16 6YD, UK

**Keywords:** cardiac arrest, resuscitation, ROSC, pre-hospital, critical care, PHEA, HEMS

## Abstract

**Background/Objectives**: Out-of-hospital cardiac arrests (OHCAs) are common, with return of spontaneous circulation (ROSC) achieved in approximately 25% of patients. However, it remains unknown whether post-ROSC care delivered by a pre-hospital critical care team (CCT) improves patient outcomes. We therefore aimed to investigate this in OHCA patients admitted to our intensive care unit (ICU). **Methods**: In this retrospective observational study, consecutive adults with ROSC after non-traumatic OHCA admitted to our ICU between 1 September 2019 and 31 August 2022 were included. We compared patients who received post-ROSC care from a CCT to those who received standard care. The primary outcome was a good neurological outcome on hospital discharge (defined as Cerebral Performance Category 1–2). Descriptive statistics, Area Under the Receiver Operating Characteristic Curve (AUC) values, and adjusted Odds Ratios (ORs) are reported. We constructed multivariable logistic regression models that adjusted for the component variables of the MIRACLE2 score. **Results**: We included 126 OHCAs (median age 63 years, 69% male), which were largely witnessed (82%), involved bystander cardiopulmonary resuscitation (87%), and had an initial shockable rhythm (61%). The prevalence of good neurological outcomes was higher in patients who received post-ROSC care from a pre-hospital CCT (37% vs. 17%, *p* = 0.012). The MIRACLE2 score was a strong predictor of good neurological outcomes (AUC 0.932), and in our multivariable analysis, good neurological outcome was associated with both CCT presence post-ROSC (aOR 3.77, 95% CI 1.02–13.89) and the delivery of PHEA (aOR 4.10, 95% CI 1.10–15.27, *p* = 0.035). Furthermore, in patients meeting the Utstein criteria (*n* = 69), good neurological outcomes were also more prevalent with CCT presence post-ROSC (62% vs. 29%, *p* < 0.001). **Conclusions**: We found that post-ROSC care delivered by a pre-hospital CCT was associated with good neurological outcomes on hospital discharge.

## 1. Introduction

Out-of-hospital cardiac arrests (OHCAs) are common and associated with poor clinical outcomes [1]. Despite extensive research, interventions that improve neurological outcomes following an OHCA are limited [2,3,4]. In the United Kingdom, cardiopulmonary resuscitation (CPR) and advanced life support (ALS) achieve return of spontaneous circulation (ROSC) in around 25% of OHCAs [5]. There are several factors contribute to a positive outcome after an OHCA, and broadly, these are related to factors during CPR and the availability of post-ROSC care. Following resuscitation, patients who achieve ROSC are often comatose and hemodynamically unstable [6]. Consequently, they require immediate respiratory and cardiovascular stabilisation during the prehospital period, followed by admission to an intensive care unit (ICU) for the continuation of organ support measures and neuro-prognostication.

In the United Kingdom (UK), the response to OHCAs by frontline paramedics is sometimes complemented by pre-hospital critical care teams (CCTs), which consist of pre-hospital emergency medicine physicians and critical care paramedics. Whilst CPR is ongoing, the presence of a CCT may facilitate additional interventions such as endovascular resuscitation [7,8], advanced pharmacological therapies [9], or even extra-corporeal life support [10]. Alternatively, if ROSC is achieved, CCTs can usually deliver more comprehensive immediate post-ROSC care, including drug-assisted endotracheal intubation as part of pre-hospital emergency anaesthesia (PHEA) [11], or the use of vasoactive drugs to maintain haemodynamic stability and cerebral perfusion. In addition, CCTs can help facilitate safe patient transfer, or bypass nearer hospitals to deliver post-ROSC patients directly to specialist Cardiac Arrest Centres, which may ultimately improve outcomes [12,13].

It remains unclear whether CCT or physician presence during OHCAs improves patient outcomes [14,15,16,17,18], with the most recent systematic review finding conflicting low-quality evidence [19]. Furthermore, the heterogeneity in OHCA management globally, as well as evolution in practice over recent years, means the generalisability of historic findings from other healthcare systems is limited. To our knowledge, no studies have investigated the question of whether a CCT improves outcomes specifically in post-ROSC patients, who are a different population to patients in cardiac arrest with ongoing CPR. As a result, CCT dispatch to OHCAs and post-ROSC delivery of PHEA have both recently been identified in the UK as top research priorities [20]. This significant knowledge gap regarding whether the care provided by the CCT during the post-resuscitation period influences outcomes in ROSC patients admitted to intensive care requires further evaluation.

Therefore, in this study, we aimed to investigate the effect of CCT delivered post-ROSC care on OHCA outcomes in the patients admitted to our ICU. Our primary objective was to assess whether the post-ROSC presence of a CCT was associated with good neurological outcome. Our secondary objective was to investigate whether delivery of PHEA had any similar association.

## 2. Materials and Methods

### 2.1. Study Design and Setting

In this single-centre retrospective observational study, we included consecutive adults admitted to our ICU with ROSC after non-traumatic OHCA between 1 September 2019 and 31 August 2022. University Hospital Southampton NHS Foundation Trust is a large tertiary hospital which functions as a Cardiac Arrest Centre [12], although there is currently no extra-corporal life support (ECLS) provision for OHCAs in our hospital. Our hospital serves a population of 1.9 million people in south central England, with the hospital itself located in the city of Southampton and surrounded by rural areas. The majority of the region is ethnically white and economically wealthy compared to the United Kingdom average. There are nine other smaller hospitals each with general intensive care units in our healthcare network, but variable levels of primary angioplasty provision.

In our region, South Central Ambulance Service is the statutory ambulance service and dispatches paramedics as the initial response to any OHCA. These paramedics deliver ALS whilst CPR is ongoing (including the routine use of supraglottic airway devices), but are limited in their post-ROSC scope of practice, being unable to provide sedation, advanced airway management, or vasoactive drugs. Hampshire & Isle of Wight Air Ambulance and Dorset & Somerset Air Ambulance are the two CCTs which provide pre-hospital critical care for some OHCA patients admitted to our hospital. These services routinely dispatch CCTs (consisting of critical care paramedics and physicians) to OHCAs which are capable of providing PHEA (defined as use of sedation and/or paralysis to facilitate post-ROSC endotracheal intubation), vasoactive drugs by bolus or infusion, intra-arterial blood pressure monitoring, and other critical care interventions, not including ECLS.

### 2.2. Data Collection

Our inclusion criteria were intubated adults who were admitted to our ICU for post-ROSC care after non-traumatic OHCAs. We excluded patients with incomplete data and those who were secondary inter-hospital transfers. Anonymised patient data were retrieved from our electronic patient record (MetaVision, iMDsoft, Tel Aviv, Israel), into which pre-hospital notes are routinely scanned and stored. The data collected included patient demographic, OHCA characteristics, and post-ROSC characteristics. We defined ‘no flow’ time as time of OHCA onset (time of 999 calls if witnessed OHCA, or estimated time of onset if unwitnessed) to CPR commencing, and ‘low flow’ time as duration of CPR until ROSC or death. The primary outcome reported was good neurological outcome on hospital discharge (defined as Cerebral Performance Category 1–2). The secondary outcome was PaCO_2_ from point of care blood tests taken immediately on emergency department arrival. MIRACLE2 is a previously validated prognostic score for OHCAs [21], which incorporates seven variables (age, witnessed OHCA, initial shockable rhythm, changing rhythm, adrenaline use, pH, pupillary light reflex), and we calculated this score on emergency department arrival for each patient.

### 2.3. Statistical Analysis

Our data are reported using conventional descriptive statistics, with categorical data presented as numbers (percentage). We used the Kolmogorov–Smirnov test to assess continuous data for normality, and as our dataset was generally non-normally distributed, present continuous variables as median (inter-quartile range; IQR). We conducted a cohort analysis to compare patients who received CCT delivered post-ROSC to those who received standard post-ROSC care. The Mann-Whitney U and Chi-square tests are used to compare continuous and categorical variables, respectively. Area Under the Receiver Operating Characteristic Curve (AUC) values are also reported. Multivariable logistic regression models, incorporating the component variables of the MIRACLE2 score (age, witnessed OHCA, initial rhythm, changing rhythm, adrenaline use, pH, pupillary light reflex) as well as CCT presence post-ROSC or delivery of PHEA, were constructed to investigate associations further, with adjusted Odds Ratios (aOR) reported. We chose to adjust for the component variables of the MIRACLE2 score as this is highly predictive of OHCA outcomes [21,22,23]. We conducted two sub-group analyses, firstly in patients meeting the Utstein criteria (witnessed OHCAs with an initial shockable rhythm), and secondly, excluding patients for whom a CCT arrived before ROSC (i.e., comparing standard care to patients who received pre-hospital critical care only post-ROSC). When necessary, pH and PaCO_2_ were dichotomized to clinically meaningful thresholds. We used SPSS v26 (IBM Corp., Armonk, NY, USA) and MedCalc v22 (MedCalc Software, Ostend, Belgium) for our analysis, with *p* < 0.05 taken as statistically significant.

### 2.4. Ethical and Research Approval

This was part of a larger cohort study investigating outcomes for critically ill patients admitted to our ICU. The study was sponsored by the University Hospital Southampton NHS Foundation Trust (RHM CRI 0370) and approved by Health Research Authority and Health and Care Research Wales (IRAS 232922). The manuscript complies with the STROBE guidelines [24].

## 3. Results

**Figure 1:**
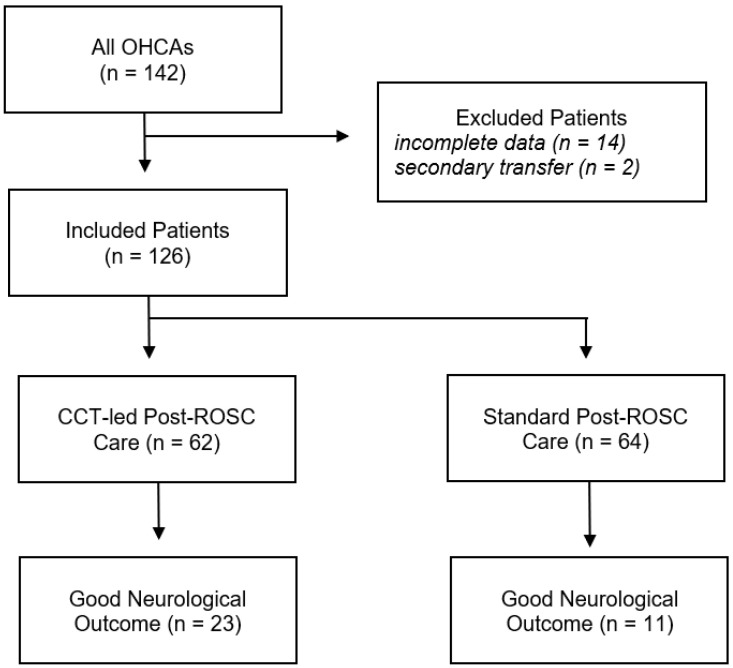
Flow diagram of eligible, included, and excluded patients, with details of the presence of CCTs and neurological outcomes.

Of 142 eligible patients admitted to our ICU during the study period, 14 were excluded due to incomplete data and 2 were secondary inter-hospital transfers (Figure 1).

### 3.1. Patient Characteristics

We included 126 patients in our analysis (Table 1). The median age was 63 years (IQR 50–74), whilst 87 patients (69%) were male. OHCAs were mainly witnessed (n = 103, 82%) with bystander CPR (n = 110, 87%), and the majority had an initially shockable rhythm (n = 77, 61%). There were 69 patients (55%) in the Utstein criteria sub-group. The median ‘no flow’ and ‘low flow’ times were 0 min (IQR 0–5) and 25 min (IQR 18–34), respectively.

After ROSC, 74 patients (59%) had reactive pupils whilst 43 patients (34%) had spontaneous respiratory effort. In 62 patients (49%), a CCT were present to provide post-ROSC care, which included both delivery of PHEA in 52 patients (41%) and pre-hospital vasoactive drugs use in 41 patients (33%). On emergency department admission, median pH was 7.16, whilst when an arterial blood sample was also obtained (n = 97), the median PaCO_2_ was 7.4 kPa (6.2–9.4). The median MIRACLE2 score was 5 (2–6), and 34 patients (27%) had a good neurological outcome on hospital discharge.

### 3.2. Predictors of Good Neurological Outcome

We compared between patients with good and poor neurological outcomes (Supplementary Table S1). In those with good neurological outcomes, more OHCAs were witnessed (97% vs. 76%, *p* = 0.007) or had an initial shockable rhythm (92% vs. 50%, *p* < 0.001). Furthermore, in patients with poor neurological outcomes, we observed a higher prevalence of ‘changing rhythms’ (50% vs. 12%, *p* < 0.001) and adrenaline use (89% vs. 24%, *p* < 0.001), as well as longer ‘no flow’ and ‘low flow’ times. Furthermore, patients with good neurological outcomes had more favourable pH (7.29 vs. 7.11, *p* = 0.013), PaCO_2_ (6.1 vs. 8.1 kPa, *p* < 0.001) and MIRACLE 2 score (1 vs. 5, *p* < 0.001) on hospital arrival.

**Figure 2:**
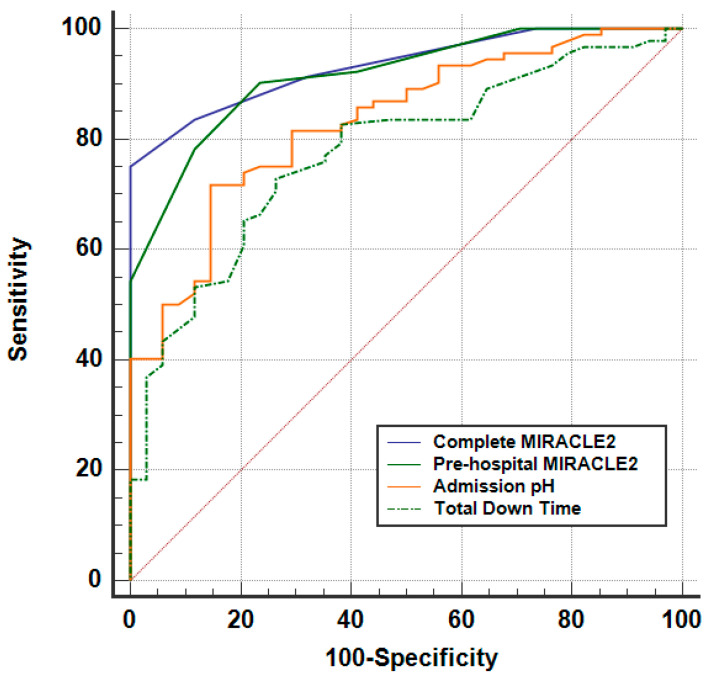
AUROC analysis for variables predictive of good neurological outcome.

We further investigated the ability of variables to predict good neurological outcome (Figure 2). MIRACLE2 score was a very strong predictor (AUC 0.932, 95% CI 0.892–0.973, *p* < 0.001), followed by a modified ‘pre-hospital’ MIRACLE2 score which excluded pH (AUC 0.915, 0.866–0.964, *p* < 0.001). Furthermore, admission pH (AUC 0.832, 95% CI 0.757–0.906, *p* < 0.001) and total time in cardiac arrest (AUC 0.778, 95% CI 0.692–0.863, *p* < 0.001) also robustly predicted good neurological outcome.

### 3.3. Effect of CCT Presence

We compared patients with CCT presence post-ROSC to those who received standard care (Table 1). OHCAs with CCT presence post-ROSC were younger patients (60 vs. 69 years, *p* = 0.003), with a higher prevalence of bystander CPR (94% vs. 81%, *p* = 0.038), and had shorter ‘no flow’ times (0 vs. 2 min, *p* = 0.036). Furthermore, when CCT was present post-ROSC, most patients received PHEA (n = 52, 84%) and pre-hospital vasoactive drugs (n = 41, 66%), whilst these interventions were not provided to any patients as part of standard ALS post-ROSC care. Of note, patients with CCT presence post-ROSC had higher survival with good neurological outcome (37% vs. 17%, *p* = 0.012), without any statistically significant difference in MIRACLE2 score (4 vs. 5, *p* = 0.131).

In our multivariable logistic regression model, CCT presence post-ROSC was independently associated with good neurological outcome (aOR 5.04, 95% CI 1.25–20.40, *p* < 0.023). Furthermore, patient age, initial shockable rhythm, adrenaline use, and pH < 7.20 were also associated with good neurological outcome (Table 2). However, there was no statistically significant association between CCT presence and a PaCO_2_ < 6 kpa on hospital arrival (aOR 3.39, 95% CI 0.94–12.25, *p* = 0.062).

We conducted two sub-group analyses. In patients meeting the Utstein criteria (n = 69), good neurological outcomes were more prevalent in patients with CCT presence post-ROSC (62% vs. 29%, *p* < 0.001). In a second sub-group (n = 105), we excluded patients with CCT presence as CPR was ongoing, leaving only patients who received standard care compared to patients for whom CCTs arrived post-ROSC. The prevalence of good neurological outcome was higher in patients with CCT presence only post-ROSC (49% vs. 17%, *p* < 0.001). Furthermore, the same multivariable model found CCT presence post-ROSC remained independently associated with good neurological outcome (aOR 5.93, 95% CI 1.38–25.42, *p* = 0.017).

### 3.4. Effect of PHEA

We compared patients who received PHEA to those managed with standard care prior to hospital admission (Supplementary Table S2). Those who received PHEA had lower MIRACLE2 scores (4 vs. 5, *p* = 0.009), lower PaCO_2_ on emergency department admission (7.3 vs. 7.9 kPa, *p* = 0.045), and a higher prevalence of good neurological outcomes (42 vs. 16%, *p* = 0.001). Furthermore, in our multivariable analysis (Table 3), delivery of PHEA was also independently associated with good neurological outcome (aOR 4.10, 95% CI 1.10–15.27, *p* = 0.035). However, there was no statistically significant association between delivery of PHEA and a PaCO_2_ < 6 kPa on emergency department arrival (aOR 3.14, 95% CI 0.92–10.72, *p* = 0.067).

## 4. Discussion

In this single-centre retrospective observational study of non-traumatic OHCAs, we found that post-ROSC care delivered by a pre-hospital CCT was associated with good neurological outcomes on hospital discharge. This association holds true in our multivariable analysis, which adjusted for widely recognised confounding variables such as patient age and initial rhythm. Although the available evidence has generally failed to demonstrate that CCT presence is associated with improved OHCA outcomes [14,15,16,17,18], to our knowledge, we are the first to investigate this question specifically in patients receiving post-ROSC care. These patients have different and more nuanced clinical needs than those in active cardiac arrest, and we hypothesise that many of the skills and capabilities of CCTs can be best utilised in a post-ROSC setting. We believe our findings are hypothesis-generating and should be investigated further in prospective or multi-centre studies.

Our data suggest that CCT presence post-ROSC is associated with good neurological outcomes, although the specific mechanisms that could explain this benefit remain unclear. One possible explanation is that experienced pre-hospital physicians and critical care paramedics can support leadership and decision-making post-ROSC, although this is difficult to quantify or study in practice. There is some evidence that the transfer of OHCA survivors directly to Cardiac Arrest Centers may improve outcomes [12,13], and a low MIRACLE2 score may help guide clinicians to make decisions about the direction of care. We and previous authors have shown that MIRACLE2 is highly predictive of OHCA outcomes [21,22,23], whilst our data also highlight how a modified ‘pre-hospital’ MIRACLE2 score (without the pH component) performs nearly as robustly. These finding may give pre-hospital clinicians the confidence to use MIRACLE2 scores as a tool to further guide post-ROSC care and patient destination.

We found the delivery of PHEA was also independently associated with good neurological outcome. PHEA is a core CCT intervention that was routinely delivered to patients in our study, and is traditionally thought to facilitate ventilation, neuroprotection, and safe transfer. Although recent European data suggest that the provision of PHEA and vasoactive drugs by a CCT may facilitate better control of post-ROSC physiological derangement [25], we did not find a statistically significant association between PHEA and favourable PaCO_2_. Tracheal intubation whilst CPR is ongoing has not been shown to improve neurological outcome [26], but in contrast to our data, other studies have found intubation is associated with improved PaCO_2_ control after ROSC [27,28]. Of note, in addition to tracheal intubation, PHEA as a therapeutic package also typically includes sedation, neuromuscular blockade, and management of ventilation by a CCT. There is little evidence that sedation improves outcomes after OHCA, but some data suggest that the CCTs are better suited to manage post-ROSC agitation or seizures [25]. Furthermore, it is increasingly appreciated that appropriate ventilator management is associated with improved long-term OHCA outcomes [29,30], and we hypothesise that pre-hospital CCTs may be more experienced at delivering this.

### Strength and Limitations

This is the first study to specifically investigate the effect of pre-hospital CCTs in patients receiving post-ROSC care after OHCAs. Our data demonstrate a possible benefit to CCT presence post-ROSC, and we believe these findings help guide OHCAs care in the UK and similar healthcare systems. However, our study has several limitations. As a single-center retrospective study in a large Cardiac Arrest Centre, our sample size was limited, and these findings may not be generalisable to other areas. The CCTs in our area have selectively dispatch to OHCAs with a perceived favourable prognosis (e.g., witnessed collapse in younger patient), although we have adjusted for the main confounding variables in our multivariable models. In addition, the difficulty of retrospective data collection means that we are unable to report CCT composition during the course of patient care, but some patients were likely managed by critical care paramedics alone initially, prior to the arrival of a physician-staffed CCT resource. Furthermore, only patients admitted to our ICU were included in our analysis, with the exclusion of OHCA patients who died prior to this (e.g., in the Emergency Department) likely to represent a further selection bias. We also do not account for in-hospital factors that could affect neurological outcomes, and, as our multivariable models adjusted for only the component variables of MIRACLE2 score, it remains possible our results were affected by unmeasured or residual confounding (e.g., aetiology). Finally, we could not accurately record the professional background of CCTs that attended patients in this study, so are unable to comment on whether physician presence was intrinsic to our findings, and we unable to follow patients beyond hospital discharge.

## 5. Conclusions

In this retrospective observational study of non-traumatic OHCA patients, post-ROSC care by a pre-hospital CCT and the delivery of PHEA were both associated with good neurological outcomes on hospital discharge. However, these findings are drawn from retrospective single-centre data, and requirement validation in multi-centre or prospective studies before changes in clinical practice are implemented.

## Figures and Tables

**Table 1:** Patient demographics, OHCA characteristics, and post-ROSC care according to whether CCT was present for post-ROSC care.

	All Patients (n = 126)	CCT-led Post-ROSC Care (n = 62)	Standard Post-ROSC Care (n = 64)	Significant Difference? (*p*)
Patient Demographics				
Age (years)	63 (50–74)	60 (49–69)	69 (55–77)	0.003
Male Sex	87 (69%)	42 (68%)	45 (70%)	0.755
OHCA Characteristics				
Witnessed	103 (82%)	51 (82%)	52 (81%)	0.882
Bystander CPR	110 (87%)	58 (94%)	52 (81%)	0.038
Bystander AED use	14 (11%)	8 (13%)	6 (9%)	0.529
Shockable 1st Rhythm	77 (61%)	35 (57%)	42 (66%)	0.291
Changing Rhythm	50 (40%)	26 (42%)	24 (38%)	0.611
Adrenaline Given	90 (71%)	42 (68%)	48 (75%)	0.367
No Flow Time (minutes)	0 (0–5)	0 (0–5)	2 (0–5)	0.036
Low Flow Time (minutes)	25 (18–34)	25 (17–38)	25 (19–32)	0.853
CCT present CPR ongoing	21 (17%)	21 (34%)	0 (0%)	<0.001
Post-ROSC Characteristics				
Any Reactive Pupils	74 (59%)	38 (61%)	36 (56%)	0.566
Any Respiratory Effort	43 (34%)	17 (27%)	26 (41%)	0.166
Pre-Hospital Anaesthesia	52 (41%)	52 (84%)	0 (0%)	<0.001
Pre-Hospital Vasoactive Drugs	41 (33%)	41 (66%)	0 (0%)	<0.001
Admission Biochemistry				
pH	7.16 (7.04–7.28)	7.19 (7.06–7.28)	7.16 (7.03–7.26)	0.388
PaCO_2_ (kPa)	7.4 (6.2–9.4)	7.4 (5.9–8.5)	8.0 (6.5–9.8)	0.105
Other				
MIRACLE2 score	5 (3–6)	4 (2–6)	5 (3–6)	0.131
Good Neurological Outcome	34 (27%)	23 (37%)	11 (17%)	0.012

Abbreviations: critical care team (CCT), return of spontaneous circulation (ROSC), out-of-hospital cardiac arrest (OHCA), cardiopulmonary resuscitation (CPR), automated external defibrillator (AED).

**Table 2:** Multivariable logistic regression models incorporating CCT presence post-ROSC and component variables of MIRACLE2 score.

Variable	Adjusted Odds Ratio (95% CI, *p*)	
	Good Neurological Outcome	PaCO_2_ < 6 kpa
Age (years)	0.94 (0.89–0.99, 0.022)	0.99 (0.96–1.03, 0.753)
Witnessed OHCA	6.84 (0.49–95.00, 0.152)	1.07 (0.18–6.33, 0.944)
Initial Shockable Rhythm	6.27 (1.10–35.76, 0.039)	3.97 (0.82–19.26, 0.087)
Changing Rhythms	0.22 (0.04–1.37, 0.105)	0.94 (0.20–4.54, 0.943)
Adrenaline Given	0.13 (0.03–0.68, 0.016)	0.83 (0.16–4.39, 0.822)
pH < 7.20	0.20 (0.05–0.85, 0.029)	0.39 (0.11–1.33, 0.131)
Reactive Pupils	1.97 (0.37–1.047, 0.42)	1.38 (0.37–5.19, 0.634)
CCT presence post ROSC	5.04 (1.25–20.40, 0.023)	3.39 (0.94–12.25, 0.062)

Abbreviations: out-of-hospital cardiac arrest (OHCA), critical care team (CCT), return of spontaneous circulation (ROSC).

**Table 3:** Multivariable logistic regression models incorporating delivery of PHEA and component variables of MIRACLE2 score.

Variable	Adjusted Odds Ratio (95% CI, *p*)	
	Good Neurological Outcome	PaCO_2_ < 6 kpa
Age (years)	0.94 (0.90–0.99, 0.027)	0.99 (0.96–1.03, 0.738)
Witnessed OHCA	6.99 (0.54–91.01, 0.138)	1.13 (0.19–6.64, 0.900)
Initial Shockable Rhythm	5.69 (1.04–31.13, 0.045)	3.51 (0.75–16.54, 0.112)
Changing Rhythms	0.23 (0.04–1.42, 0.114)	1.03 (0.21–4.92, 0.973)
Adrenaline Given	0.16 (0.03–0.82, 0.028)	0.86 (0.16–4.60, 0.860)
pH < 7.20	0.21 (0.05–0.86, 0.030)	0.37 (0.11–1.28, 0.117)
Reactive Pupils	1.89 (0.36–9.87, 0.451)	1.34 (0.36–5.03, 0.666)
Delivery of PHEA	4.10 (1.10–15.27, 0.035)	3.14 (0.92–10.72, 0.067)

Abbreviations: out-of-hospital cardiac arrest (OHCA), pre-hospital emergency anaesthesia (PHEA).

## Data Availability

The datasets used and/or analysed during the current study are available from the corresponding author on reasonable request.

## Supplementary Materials

The following supporting information can be downloaded at: https://www.mdpi.com/article/10.3390/jcm14030966/s1, Table S1: Patient demographics, OHCA characteristics, and post-ROSC care according to neurological outcome.; Table S2: Patient demographics, OHCA characteristics, and post-ROSC care according to whether PHEA was delivered.

## Abbreviations

The following abbreviations are used in this manuscript:

OHCA	Out-of-Hospital Cardiac Arrest
ROSC	Return Of Spontaneous Circulation
CCT	Critical Care Team
ICU	Intensive Care Unit
AUC	Area Under the Receiver Operating Characteristic Curve
OD	Odds Ratio
CPR	Cardiopulmonary Resuscitation
ALS	Advanced Life Support
PHEA	Pre-Hospital Emergency Anaesthesia
ECLS	Extra-Corporeal Life Support
IQR	Inter-quartile Range
AED	Automated External Defibrillation

## References

[1] Nishiyama C., Kiguchi T., Okubo M., Alihodžić H., Al-Araji R., Baldi E., Beganton F., Booth S., Bray J., Christensen E. (2023). Three-year trends in out-of-hospital cardiac arrest across the world: Second report from the International Liaison Committee on Resuscitation (ILCOR). Resuscitation.

[2] Eastwood G., Nichol A.D., Hodgson C., Parke R.L., McGuinness S., Nielsen N., Bernard S., Skrifvars M.B., Stub D., Taccone F.S. (2023). Mild Hypercapnia or Normocapnia after Out-of-Hospital Cardiac Arrest. N. Engl. J. Med..

[3] Desch S., Freund A., Akin I., Behnes M., Preusch M.R., Zelniker T.A., Skurk C., Landmesser U., Graf T., Eitel I. (2021). Angiography after out-of-Hospital Cardiac Arrest without ST-Segment Elevation. N. Engl. J. Med..

[4] Dankiewicz J., Cronberg T., Lilja G., Jakobsen J.C., Levin H., Ullén S., Rylander C., Wise M.P., Oddo M., Cariou A. (2021). Hypothermia versus Normothermia after Out-of-Hospital Cardiac Arrest. N. Engl. J. Med..

[5] OHCAO n.d. https://warwick.ac.uk/fac/sci/med/research/ctu/trials/ohcao/.

[6] Nolan J.P., Sandroni C., Böttiger B.W., Cariou A., Cronberg T., Friberg H., Genbrugge C., Haywood K., Lilja G., Moulaert V.R.M. (2021). European Resuscitation Council and European Society of Intensive Care Medicine guidelines 2021, Post-resuscitation care. Intensive Care Med..

[7] Aziz S., Lachowycz K., Major R., Rees P., Barratt J. (2024). Implementation of advanced vascular access, physiological monitoring and goal-directed resuscitation during OHCA in a helicopter emergency medical service. J. Vasc. Access.

[8] Gamberini L., Coniglio C., Lupi C., Tartaglione M., Mazzoli C.A., Baldazzi M., Cecchi A., Ferri E., Chiarini V., Semeraro F. (2021). Resuscitative endovascular occlusion of the aorta (REBOA) for refractory out of hospital cardiac arrest. An Utstein-based case series. Resuscitation.

[9] Watson A., Hannah J., Owen P., Plumb J. (2024). Outcomes of a ‘de-emphasised’ adrenaline strategy for refractory ventricular fibrillation. Resusc. Plus.

[10] Low C.J.W., Ling R.R., Ramanathan K., Chen Y., Rochwerg B., Kitamura T., Iwami T., Ong M.E.H., Okada Y. (2024). Extracorporeal cardiopulmonary resuscitation versus conventional CPR in cardiac arrest: An updated meta-analysis and trial sequential analysis. Crit. Care.

[11] Hodkinson M., Poole K. (2023). Induction of pre-hospital emergency anaesthesia i-PHEA: A national survey of UK HEMS practice. BMC Emerg. Med..

[12] Boulton A.J., Abelairas-Gómez C., Olaussen A., Skrifvars M.B., Greif R., Yeung J. (2024). Cardiac arrest centres for patients with non-traumatic cardiac arrest: A systematic review. Resuscitation..

[13] Price J., Rees P., Lachowycz K., Starr Z., Pareek N., Keeble T.R., Major R., Barnard E.B. (2024). Increased survival for resuscitated Utstein-comparator group patients conveyed directly to cardiac arrest centres in a large rural and suburban population in England. Resuscitation.

[14] Shinada K., Matsuoka A., Miike T., Koami H., Sakamoto Y. (2024). Effects of physician-present prehospital care in patients with out-of-hospital cardiac arrest on return of spontaneous circulation: A retrospective, observational study in Saga, Japan. Health Sci. Rep..

[15] Hamilton A., Steinmetz J., Wissenberg M., Torp-Pedersen C., Lippert F.K., Hove L., Lohse N. (2016). Association between prehospital physician involvement and survival after out-of-hospital cardiac arrest: A Danish nationwide observational study. Resuscitation.

[16] von Vopelius-Feldt J., Coulter A., Benger J. (2015). The impact of a pre-hospital critical care team on survival from out-of-hospital cardiac arrest. Resuscitation.

[17] Olasveengen T.M., Lund-Kordahl I., Steen P.A., Sunde K. (2009). Out-of hospital advanced life support with or without a physician: Effects on quality of CPR and outcome. Resuscitation.

[18] Estner H.L., Günzel C., Ndrepepa G., William F., Blaumeiser D., Rupprecht B., Hessling G., Deisenhofer I., Weber M.A., Wilhelm K. (2007). Outcome after out-of-hospital cardiac arrest in physician-staffed emergency medical system according to the Utstein style. Am. Heart J..

[19] von Vopelius-Feldt J., Brandling J., Benger J. (2017). Systematic review of the effectiveness of prehospital critical care following out-of-hospital cardiac arrest. Resuscitation..

[20] Ramage L., McLachlan S., Williams K. (2023). PreHOspital Trainee Operated research Network (PHOTON). Determining the top research priorities in UK prehospital critical care: A modified Delphi study. Emerg. Med. J..

[21] Pareek N., Kordis P., Beckley-Hoelscher N., Pimenta D., Kocjancic S.T., Jazbec A., Nevett J., Fothergill R., Kalra S., Lockie T. (2020). A practical risk score for early prediction of neurological outcome after out-of-hospital cardiac arrest: MIRACLE2. Eur. Heart J..

[22] Schmidbauer S., Rylander C., Cariou A., Wise M.P., Thomas M., Keeble T.R., Erlinge D., Haenggi M., Wendel-Garcia P.D., Bělohlávek J. (2023). Comparison of four clinical risk scores in comatose patients after out-of-hospital cardiac arrest. Resuscitation.

[23] Aldous R., Roy R., Cannata A., Abdrazak M., Mohanan S., Beckley-Hoelscher N., Stahl D., Kanyal R., Kordis P., Sunderland N. (2023). MIRACLE2 Score Compared With Downtime and Current Selection Criterion for Invasive Cardiovascular Therapies After OHCA. JACC Cardiovasc. Interv..

[24] Von Elm E., Altman D.G., Egger M., Pocock S.J., Gøtzsche P.C., Vandenbroucke J.P. (2008). The Strengthening the Reporting of Observational Studies in Epidemiology (STROBE) statement: Guidelines for reporting observational studies. J. Clin. Epidemiol..

[25] Vos I., Lucassen F., Bens B., Dercksen B., Postma R., Jorna E., ter Maaten J., Struys M., ter Avest E. (2024). Pre-hospital care after return of spontaneous circulation: Are we achieving our targets?. Resusc. Plus.

[26] Benger J.R., Kirby K., Black S., Brett S.J., Clout M., Lazaroo M.J., Nolan J.P., Reeves B.C., Robinson M., Scott L.J. (2018). Effect of a Strategy of a Supraglottic Airway Device vs Tracheal Intubation During Out-of-Hospital Cardiac Arrest on Functional Outcome: The AIRWAYS-2 Randomized Clinical Trial. JAMA.

[27] Nakayama R., Bunya N., Uemura S., Sawamoto K., Narimatsu E. (2024). Advanced Airway Management and Ventilation for Out-of-Hospital Cardiac Arrest with Prehospital Return of Spontaneous Circulation: A Prospective Observational Cohort Study in Japan. Prehosp Emerg. Care.

[28] Song S.R., Kim K.H., Park J.H., Song K.J., Shin S.D. (2023). Association between prehospital airway type and oxygenation and ventilation in out-of-hospital cardiac arrest. Am. J. Emerg. Med..

[29] Robba C., Badenes R., Battaglini D., Ball L., Brunetti I., Jakobsen J.C., Lilja G., Friberg H., Wendel-Garcia P.D., Young P.J. (2022). Ventilatory settings in the initial 72 h and their association with outcome in out-of-hospital cardiac arrest patients: A preplanned secondary analysis of the targeted hypothermia versus targeted normothermia after out-of-hospital cardiac arrest (TTM2) trial. Intensive Care Med..

[30] Newell C., Grier S., Soar J. (2018). Airway and ventilation management during cardiopulmonary resuscitation and after successful resuscitation. Crit. Care.

