# Supplementary material for: Pre-Hospital Critical Care for Out-of-Hospital Cardiac Arrests with Return of Spontaneous Circulation: A Retrospective Observational Study
Source: J Clin Med. 2025 Feb 3;14(3):966. doi: 10.3390/jcm14030966 (PMC11818504; doi:10.3390/jcm14030966)
Supplement: Supplementary file 1 [file jcm-14-00966-s001.zip › jcm-3436176-supplementary.pdf]

**Supplementary Table S1 – Patient demographics, OHCA characteristics, and post-ROSC care according to neurological outcome.**

| | **Good Neurological Outcome** (n = 34) | **Poor Neurological Outcome** (n = 92) | **Significant Difference?** (p) |
|---|---|---|---|
| **Patient Demographics** | | | |
| Age (years) | 58 (47 – 70) | 64 (51 – 74) | 0.141 |
| Male Sex | 24 (71%) | 63 (68%) | 0.820 |
| **OHCA Characteristics** | | | |
| Witnessed | 33 (97%) | 70 (76%) | 0.007 |
| Bystander CPR | 32 (94%) | 78 (85%) | 0.162 |
| Bystander AED use | 8 (24%) | 6 (7%) | 0.007 |
| Shockable 1st Rhythm | 31 (92%) | 46 (50%) | <0.001 |
| Changing Rhythm | 4 (12%) | 46 (50%) | <0.001 |
| Adrenaline Given | 8 (24%) | 82 (89%) | <0.001 |
| No Flow Time (minutes) | 0 (0 – 1) | 2 (0 – 6) | <0.001 |
| Low Flow Time (minutes) | 19 (15 – 25) | 29 (20 – 39) | <0.001 |
| CCT present CPR ongoing | 3 (9%) | 18 (20%) | 0.151 |
| **Post-ROSC Characteristics** | | | |
| CCT present post-ROSC | 23 (68%) | 39 (42%) | 0.012 |
| Any Reactive Pupils | 29 (86%) | 45 (49%) | <0.001 |
| Any Respiratory Effort | 21 (62%) | 22 (24%) | <0.001 |
| Pre-Hospital Anaesthesia | 22 (65%) | 30 (33%) | 0.001 |
| Pre-Hospital Vasoactive Drugs | 14 (42%) | 27 (29%) | 0.208 |
| **Admission Biochemistry** | | | |
| pH | 7.29 (7.22 - 7.32) | 7.11 (6.97 - 7.22) | 0.013 |
| $PaCO_2$ (kPa) | 6.1 (5.7 - 6.7) | 8.1 (6.9 - 10.0) | <0.001 |
| **Other** | | | |
| MIRACLE2 score | 1 (0 – 3) | 5 (5 – 6) | <0.001 |
| **Abbreviations**: out-of-hospital cardiac arrest (OHCA), return of spontaneous circulation (ROSC), critical care team (CCT), cardiopulmonary resuscitation (CPR), automated external defibrillation (AED) | | | |

**Supplementary Table S2 – Patient demographics, OHCA characteristics, and post-ROSC care according to whether PHEA was delivered.**

| | **PHEA** (n = 52) | **Standard Care** (n = 74) | **Significant Difference?** (p) |
|---|---|---|---|
| **Patient Demographics** | | | |
| Age (years) | 56 (48 – 69) | 67 (56 – 77) | 0.002 |
| Male Sex | 34 (65%) | 53 (72%) | 0.323 |
| **OHCA Characteristics** | | | |
| Witnessed | 43 (83%) | 60 (81%) | 0.818 |
| Bystander CPR | 49 (94%) | 61 (82%) | 0.050 |
| Bystander AED use | 8 (15%) | 6 (8%) | 0.201 |
| Shockable 1st Rhythm | 31 (60%) | 46 (62%) | 0.773 |
| Changing Rhythm | 20 (38%) | 30 (41%) | 0.814 |
| Adrenaline Given | 32 (62%) | 58 (78%) | 0.039 |
| No Flow Time (minutes) | 0 (0 – 4) | 2 (0 – 5) | 0.031 |
| Low Flow Time (minutes) | 21 (16 – 34) | 27 (19 – 33) | 0.246 |
| CCT present CPR ongoing | 16 (31%) | 5 (7%) | <0.001 |
| **Post-ROSC Characteristics** | | | |
| CCT present post-ROSC | 52 (100%) | 10 (14%) | <0.001 |
| Any Reactive Pupils | 34 (65%) | 40 (54%) | 0.203 |
| Any Respiratory Effort | 17 (33%) | 26 (35%) | 0.776 |
| Pre-Hospital Vasoactive Drugs | 34 (65%) | 7 (9%) | <0.001 |
| **Admission Biochemistry** | | | |
| pH | 7.21 (7.07 – 7.29) | 7.14 (6.99 – 7.25) | 0.077 |
| $PaCO_2$ (kPa) | 7.3 (5.8 – 8.1) | 8.0 (6.6 – 9.8) | 0.044 |
| **Other** | | | |
| MIRACLE2 score | 4 (1 – 6) | 5 (3 – 6) | 0.009 |
| Good Neurological Outcome | 22 (42%) | 12 (16%) | 0.001 |

**Abbreviations**: out-of-hospital cardiac arrest (OHCA), return of spontaneous circulation (ROSC), pre-hospital emergency anaesthesia (PHEA), critical care team (CCT), cardiopulmonary resuscitation (CPR), automated external defibrillation (AED)
